# Supplementary material for: A genomics learning framework for undergraduates
Source: PLoS One. 2025 Jan 9;20(1):e0313124. doi: 10.1371/journal.pone.0313124 (PMC11717232; doi:10.1371/journal.pone.0313124)
Supplement: S1 File — (PDF) [file pone.0313124.s001.pdf]

INTRODUCTION - the Genomics Concepts

Genomics is the study of the genome of a single organism or a set of organisms, and may include organelle genomes depending on context. While much has been learned about genes and the relationship between genotype and phenotype, genomics provides a more complete understanding of the polygenic nature of traits and the role that evolution plays in the development of variation, including speciation. The field of genomics is characterized by concepts derived from molecular biology and evolution, but focuses on the whole genome. Genomes include not only the sequences coding for proteins and for RNA products ("genes"), but also regulatory sequences. In the case of eukaryotes, much of the genome can be made up of repetitive elements, including transposable elements and their remnants, and how they are packaged. In contrast, prokaryotic genomes are minimal, having been stripped down to essential information by selection. In the materials that follows "genome" will mean the complex assembly of coding and non-coding information, with the notion that evolution acts on the genome as a whole.

Because genomics is becoming an increasingly valuable part of biological research, we believe it is important for life science students to become familiar with the tenets of genomics so that they are better equipped for graduate study and for entry into the workforce. Both academic and applied research fields are increasingly reliant on aspects of data science, and genomics provides a good introduction to "big data". Genomics offers wonderful opportunities for many students to engage in authentic research that can be undertaken anywhere with, at minimum, a computer and an internet connection.

To assist in implementation of a genomics curriculum, we have identified a collection of key concepts we view as central to an understanding of genomics. At this point, the Genomics Education Alliance (GEA; https://qubeshub.org/community/groups/gea) is seeking input from the community to refine these concepts. Future work will include development of curricula that feature these concepts as well assessments that measure mastery of the concepts. Simply, which of the following statements are important for an undergraduate student in Biology (broadly) to know upon graduation? If you have questions about this project, please contact Anne Rosenwald (rosenwaa@georgetown.edu), Doug Chalker (dchalker@wustl.edu), or Laura Reed (lreed1@ua.edu).

We first start with a few questions about your students and your institution.

* Required

1. I currently teach genomics in at least one class. *

Mark only one oval.

- Yes
- No

2. I currently teach genomics to first and second year undergraduates (answer yes even if only a small fraction of time is devoted to genomics).

Mark only one oval.

- Yes
- No

3. If you answered yes to the previous question, what percentage of the class is devoted to genomics?

____

4. What size is your first and second year genomics-containing class (on average)?

Mark only one oval.

- 1-15 students
- 16-30 students
- 31-60 students
- 61-150 students
- More than 150 students
- Not applicable

5. I currently teach genomics to upper-level students as part or all of a class. *

Mark only one oval.

- ( ) Yes
- ( ) No

6. If you answered yes to the previous question, what percentage of the course is devoted to genomics?

____

7. What size is your upper-level class in genomics (on average)?

Mark only one oval.

- ( ) 1-15 students
- ( ) 16-30 students
- ( ) 31-60 students
- ( ) 61-150 students
- ( ) More than 150 students
- ( ) Not applicable

8. What is the name of your institution? *

____

Biological Concepts

For each statement, indicate the extent to which you agree it should be included in the list of Genomics Concepts for undergraduate Biology majors to know upon graduation.
A complete list of the Concepts can be found here (https://docs.google.com/document/d/1nSdGcZcicxSlZryMDbBiJ9IEitr1dg5iZ9xmXton1Oc/edit?usp=sharing).
There is a text box at the end of this section, but you may also make comments on the attached list.

9. A. Phylogenetic relationships, reflecting common ancestry, inform our understanding and interpretation of genomic information.

Mark only one oval.

	1	2	3	4	5	
Not	◯	◯	◯	◯	◯	Extremely important to include

10. A 1. Evolutionary forces such as mutation, selection, recombination, and population genetics (including neutral evolution) influence properties and patterns of genome sequence and organization within and between species. The divergence between groups under study is a factor to be considered depending on the research question (examples: divergence of protein coding sequences, divergence of regulatory sequences).

Mark only one oval.

	1	2	3	4	5	
Not	◯	◯	◯	◯	◯	Extremely important to include

11. A 2. The evolving genome is a complex of DNA and the other molecules (largely proteins) tha package it within the nucleus and/or cell. In eukaryotes, this packaging is via nucleosomes.

Mark only one oval.

	1	2	3	4	5	
Not	◯	◯	◯	◯	◯	Extremely important to include

12. A 3. Comparison of multiple genomes provides insight into evolutionary forces acting broadly, including processes like de novo gene birth, gene duplication, and sub-functionalization.

Mark only one oval.

	1	2	3	4	5	
Not	◯	◯	◯	◯	◯	Extremely important to include

13. A 4. Genomic traits can be shared or distinct among taxonomic groups and can be used to indicate evolutionary relationships across life. Taxa that share a distinctive genomic feature likely share that feature through an identifiable evolutionary mechanism (e.g., common ancestry, horizontal gene transfer).

Mark only one oval.

	1	2	3	4	5	
Not	◯	◯	◯	◯	◯	Extremely important to include

14. A 5. Comparative genomics has provided new insights into structure and function of genes. *

Mark only one oval.

	1	2	3	4	5	
Not	◯	◯	◯	◯	◯	Extremely important to include

15. B. Individual regions of a genome are expected to have different functions as a result of sequence differences. In eukaryotes, functional states can be determined in addition by DNA packaging into alternative chromatin states, and switched by changes in chromatin structure.

Mark only one oval.

	1	2	3	4	5	
Not	◯	◯	◯	◯	◯	Extremely important to include

16. C. Genomes contain both coding and non-coding regions (non-coding regions include origins of replication, and for eukaryotic genomes: centromeres, telomeres, repetitive DNA, in addition); the functions (if any) of many of the non-coding regions remain to be determined. Nonetheless, the presence of this DNA in the genome will have an impact, in addition to supplying raw material for evolution.

Mark only one oval.

	1	2	3	4	5	
Not	◯	◯	◯	◯	◯	Extremely important to include

17. D. Genomes contain information that determines both temporal and spatial patterns of gene expression and the response to environmental conditions.

Mark only one oval.

	1	2	3	4	5	
Not	◯	◯	◯	◯	◯	Extremely important to include

18. D 1. Gene expression includes transcription of products that function as an RNA molecule (tRNA, rRNA, lncRNA, miRNA, snoRNA, etc.). We have acquired a deeper understanding of such molecules as a result of genomics.

Mark only one oval.

	1	2	3	4	5	
Not	◯	◯	◯	◯	◯	Extremely important to include

19. D 2. Gene expression also includes transcription to RNA, followed by processing into mRNA, which is then translated to protein. In eukaryotes, related proteins may result from alternative splicing of the transcript.

Mark only one oval.

	1	2	3	4	5	
Not	◯	◯	◯	◯	◯	Extremely important to include

20. D 3. Biochemical organization, including nucleosome packaging, of the genome is a major determinant of gene expression.

Mark only one oval.

	1	2	3	4	5	
Not	◯	◯	◯	◯	◯	Extremely important to include

21. D 4. For a given gene and its potential protein product, expression is regulated at the transcriptional, post-transcriptional, translational, and/or post-translational levels. This can include covalent modification of RNA and protein.

Mark only one oval.

	1	2	3	4	5	
Not	◯	◯	◯	◯	◯	Extremely important to include

22. E. Genomes exhibit sequence variation (SNPs, etc.) and structural variation (rearrangements, copy number variation, transposable elements) within and between specie that can lead to differences in form and function.

Mark only one oval.

	1	2	3	4	5	
Not	◯	◯	◯	◯	◯	Extremely important to include

23. E 1. Phenotype is in part determined by patterns of gene expression that vary between individuals and between species as a result of environmental cues.

Mark only one oval.

	1	2	3	4	5	
Not	◯	◯	◯	◯	◯	Extremely important to include

24. E 2. Not all features of genes and genomes (introns, promoters, stop codons, genetic codes) are common to all species. This includes organelle genomes.

Mark only one oval.

	1	2	3	4	5	
Not	◯	◯	◯	◯	◯	Extremely important to include

25. E 3. Eukaryotic genomes are distinctive in their capacity to retain both active copies and remnants of transposable elements (TEs), which can be the majority of the DNA. Active TEs can be mutagens; thus, TEs are mostly targeted for silencing by the host. However, TEs can be activated by stress, and may be a driving force in genome evolution.

Mark only one oval.

	1	2	3	4	5	
Not	◯	◯	◯	◯	◯	Extremely important to include

26. E 4. Prokaryotic genomes acquire and lose genes as a result of horizontal gene transfer, which provides drivers for evolution.

Mark only one oval.

	1	2	3	4	5	
Not	◯	◯	◯	◯	◯	Extremely important to include

27. E 5. Viruses (including bacteriophages) have genomes that operate within their hosts and ca[illegible]
have distinct evolutionary patterns that provide regulatory constraints on their parasitic lifestyle.

Mark only one oval.

	1	2	3	4	5	
Not	◯	◯	◯	◯	◯	Extremely important to include

28. E 6. Genomic information generates new ethical, medical, and societal challenges and opportunities.

Mark only one oval.

	1	2	3	4	5	
Not	◯	◯	◯	◯	◯	Extremely important to include

29. Which of these concepts do you teach in your first and second year courses?

Check all that apply.

☐ A. Phylogenetic relationships, reflecting common ancestry, inform our understanding and interpretation of genomic information.

☐ B. Individual regions of a genome are expected to have different functions as a result of seque[illegible] differences. In eukaryotes, functional states can be determined in addition by DNA packaging into alternative chromatin states, and switched by changes in chromatin structure.

☐ C. Genomes contain both coding and non-coding regions (non-coding regions include origins o[illegible] replication, and for eukaryotic genomes: centromeres, telomeres, repetitive DNA, in addition); the functions (if any) of many of the non-coding regions remain to be determined. Nonetheless, the presence of this DNA in the genome will have an impact, in addition to supplying raw material for evolution.

☐ D. Genomes contain information that determines both temporal and spatial patterns of gene expression and the response to environmental conditions.

☐ E. Genomes exhibit sequence variation (SNPs, etc.) and structural variation (rearrangements, c[illegible] number variation, transposable elements) within and between species that can lead to differences [illegible] form and function.

30. Which of these concepts do you teach in your upper level courses?

Check all that apply.

- [ ] A. Phylogenetic relationships, reflecting common ancestry, inform our understanding and interpretation of genomic information.
- [ ] B. Individual regions of a genome are expected to have different functions as a result of sequer differences. In eukaryotes, functional states can be determined in addition by DNA packaging into alternative chromatin states, and switched by changes in chromatin structure.
- [ ] C. Genomes contain both coding and non-coding regions (non-coding regions include origins o replication, and for eukaryotic genomes: centromeres, telomeres, repetitive DNA, in addition); the functions (if any) of many of the non-coding regions remain to be determined. Nonetheless, the presence of this DNA in the genome will have an impact, in addition to supplying raw material for evolution.
- [ ] D. Genomes contain information that determines both temporal and spatial patterns of gene expression and the response to environmental conditions.
- [ ] E. Genomes exhibit sequence variation (SNPs, etc.) and structural variation (rearrangements, c number variation, transposable elements) within and between species that can lead to differences i form and function.

31. Comments about the Biological Concepts, including suggestions for other biological concepts that don't appear here and should.

____

____

____

____

____

Methodological Concepts

For each statement, indicate the extent to which you agree it should be included in the list of Genomics Concepts.
A complete list of the Concepts can be found here (https://docs.google.com/document/d/1nSdGcZcicxSlZryMDbBiJ9IEitr1dg5iZ9xmXton1Oc/edit?usp=sharing).
There is a text box at the end of this section, but you may also make comments on the attached list.

32. A. Genome-scale studies have statistical and experimental design considerations that impact their accuracy. Consideration needs to be given to sample size, biological replication, technical replication, and study design.

Mark only one oval.

	1	2	3	4	5	
Not	◯	◯	◯	◯	◯	Extremely important to include

33. B. Sequencing is never completely accurate and hence models of any particular species' genome may change with new data. The methods used to gather genomic data influence dat quality and data processing strategies, which impact the final model and hence our concepts of how the genome functions. Nevertheless, new technologies have made it now possible to gather more genomic information from a wider range of species than ever before.

Mark only one oval.

	1	2	3	4	5	
Not	◯	◯	◯	◯	◯	Extremely important to include

34. C. Improvements in technology have greatly improved the speed of acquiring genomics data, and the accuracy of that data; this includes improvements in DNA sequencing, RNA sequencing/mapping, and chromatin structure mapping.

Mark only one oval.

	1	2	3	4	5	
Not	◯	◯	◯	◯	◯	Extremely important to include

35. D. Genomic analyses provide information on a population level scale that permits deeper understanding of prevalent allele frequencies, including disease alleles.

Mark only one oval.

	1	2	3	4	5	
Not	◯	◯	◯	◯	◯	Extremely important to include

36. E. Metagenomics analyses using deep sequencing has led to a better understanding of environmental niches.

Mark only one oval.

	1	2	3	4	5	
Not	◯	◯	◯	◯	◯	Extremely important to include

37. F. Computational algorithms generate predictions about the presence and detailed structure [illegible] genes, molecular function of the gene products, and common ancestry of genomic regions. [illegible] most cases, human analysis of the data can improve the accuracy of the predictions.

Mark only one oval.

	1	2	3	4	5	
Not	◯	◯	◯	◯	◯	Extremely important to include

38. G. Genomics is an application of Data Science. *

Mark only one oval.

	1	2	3	4	5	
Not	◯	◯	◯	◯	◯	Extremely important to include

39. Which of these concepts do you teach in your first and second year courses?

Check all that apply.

- [ ] A. Genome-scale studies have statistical and experimental design considerations that impact their accuracy. Consideration needs to be given to sample size, biological replication, technical replication, and study design.
- [ ] B. Sequencing is never completely accurate and hence models of any particular species' genon may change with new data. The methods used to gather genomic data influence data quality and da processing strategies, which impact the final model and hence our concepts of how the genome functions. Nevertheless, new technologies have made it now possible to gather more genomic information from a wider range of species than ever before.
- [ ] C. Improvements in technology have greatly improved the speed of acquiring genomics data, ar the accuracy of that data; this includes improvements in DNA sequencing, RNA sequencing/mappir and chromatin structure mapping.
- [ ] D. Genomic analyses provide information on a population level scale that permits deeper understanding of prevalent allele frequencies, including disease alleles.
- [ ] E. Metagenomics analyses using deep sequencing has led to a better understanding of environmental niches.
- [ ] F. Computational algorithms generate predictions about the presence and detailed structure of genes, molecular function of the gene products, and common ancestry of genomic regions. In mos cases, human analysis of the data can improve the accuracy of the predictions.
- [ ] G. Genomics is an application of Data Science.

40. Which of these concepts do you teach in your upper level courses?

Check all that apply.

- [ ] A. Genome-scale studies have statistical and experimental design considerations that impact their accuracy. Consideration needs to be given to sample size, biological replication, technical replication, and study design.
- [ ] B. Sequencing is never completely accurate and hence models of any particular species' genon may change with new data. The methods used to gather genomic data influence data quality and d processing strategies, which impact the final model and hence our concepts of how the genome functions. Nevertheless, new technologies have made it now possible to gather more genomic information from a wider range of species than ever before.
- [ ] C. Improvements in technology have greatly improved the speed of acquiring genomics data, a the accuracy of that data; this includes improvements in DNA sequencing, RNA sequencing/mappi and chromatin structure mapping.
- [ ] D. Genomic analyses provide information on a population level scale that permits deeper understanding of prevalent allele frequencies, including disease alleles.
- [ ] E. Metagenomics analyses using deep sequencing has led to a better understanding of environmental niches.
- [ ] F. Computational algorithms generate predictions about the presence and detailed structure of genes, molecular function of the gene products, and common ancestry of genomic regions. In mos cases, human analysis of the data can improve the accuracy of the predictions.
- [ ] G. Genomics is an application of Data Science.

41. Comments about the Methodological Concepts, including suggestions for other methodologic concepts that don't appear here and should.

____

____

____

____

____

42. Thank you for your input into this project. If there are other comments you would like to make please write them here.

This content is neither created nor endorsed by Google.

Google Forms
