# Supplementary figures and images for: A genomics learning framework for undergraduates
Source: PLoS One. 2025 Jan 9;20(1):e0313124. doi: 10.1371/journal.pone.0313124 (PMC11717232; doi:10.1371/journal.pone.0313124)

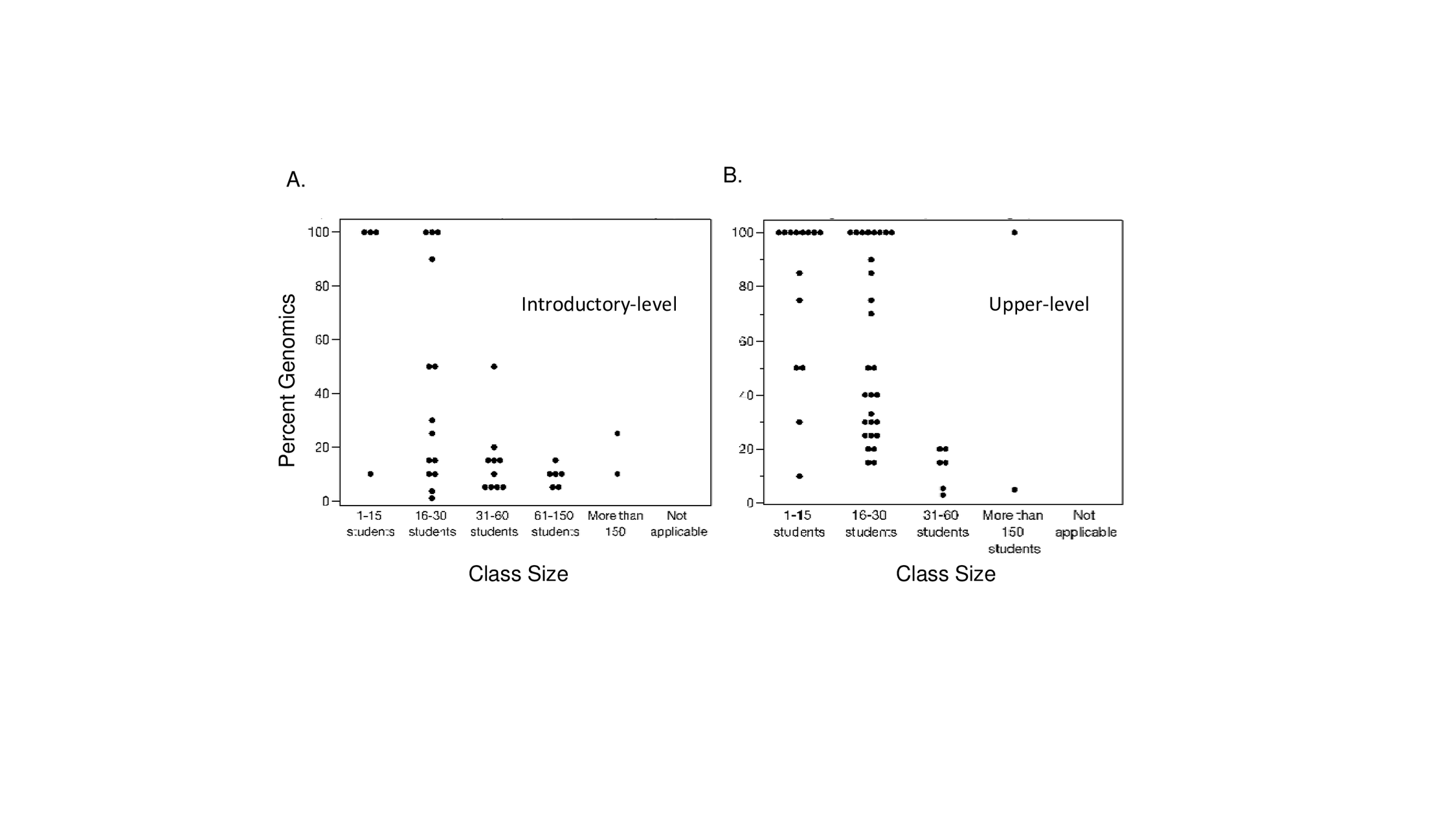

Supplement: S1 Fig — Introductory (A) and Upper-level (B) courses tended to contain more genomics when they were small, and class size tended to decrease in size at the upper relative to the introductory level. (TIF) [file pone.0313124.s002.tif]

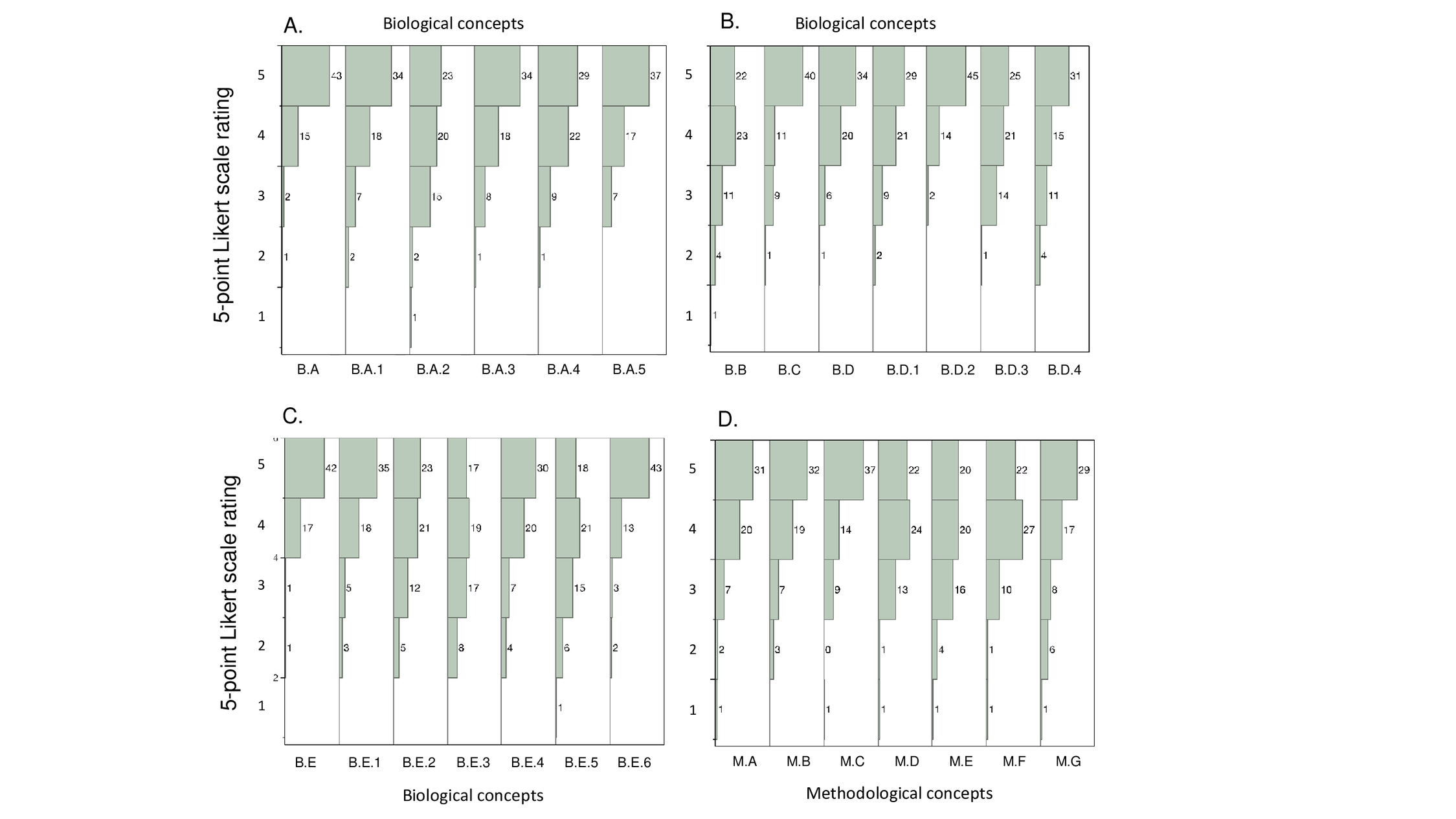

Supplement: S2 Fig — Initial concepts are provided in S1 Table. A. Initial biological concept A and subparts (A1-A5). B. Initial biological concepts B-D and subparts (D1-D4). C. Initial biological concepts E and subparts (E1-E6). D. Initial methodological concepts A-G. (TIF) [file pone.0313124.s003.tif]

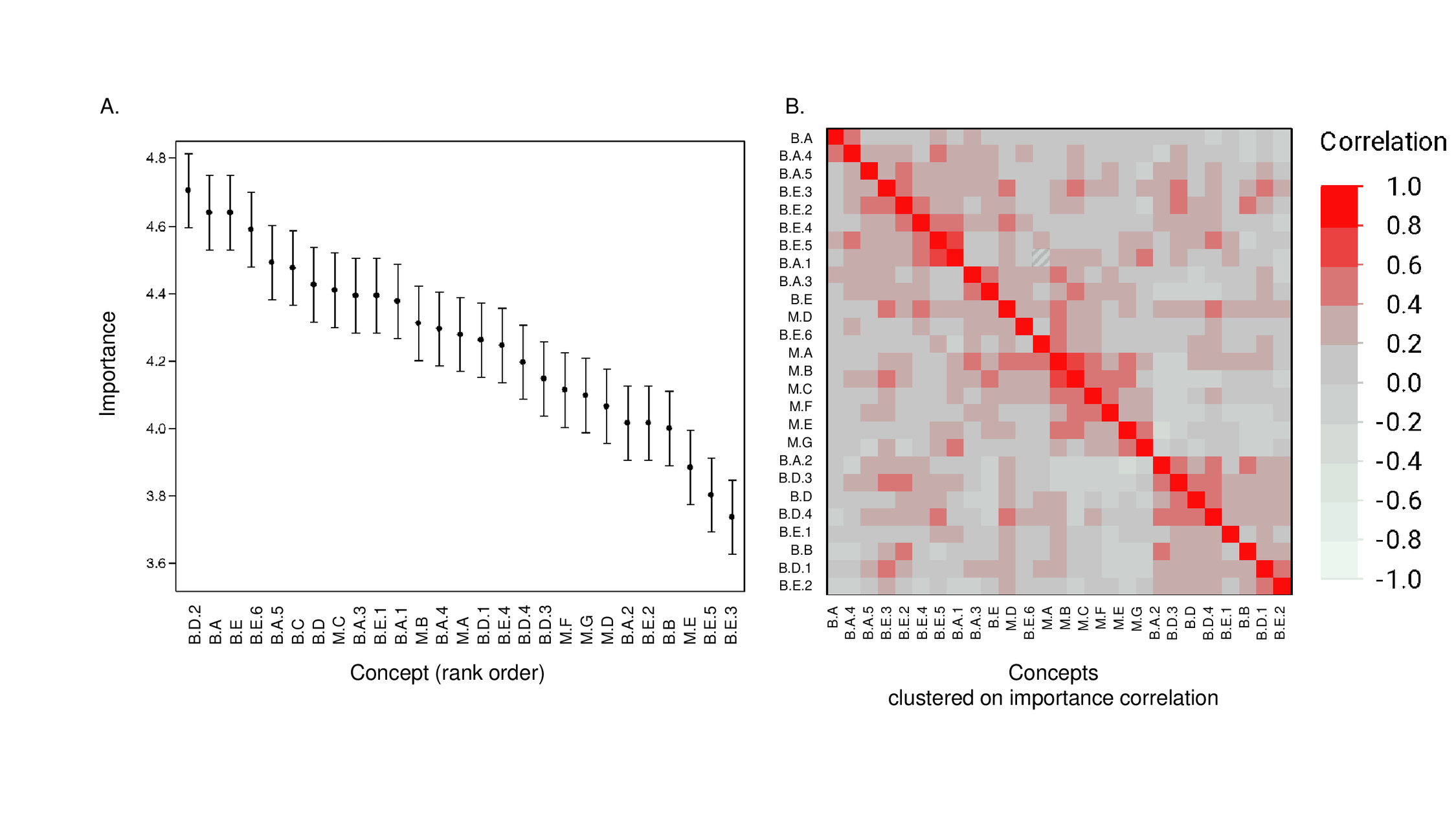

Supplement: S3 Fig — B.# = biological concept, M.# = methodological concept, as listed in S1 Table. A. Average importance scores (+/- 1SE) in rank order from most to least important. All concepts were scored as being moderately to highly important (>3.73), with biological concepts generally ranked more highly. B. Correlations of importance scores for initial concepts clustered based on correlation. Methodological concept scores tended to correlate with other methodology concept scores, and biological importance scores tended to correlate with other biological concept scores. (TIF) [file pone.0313124.s004.tif]

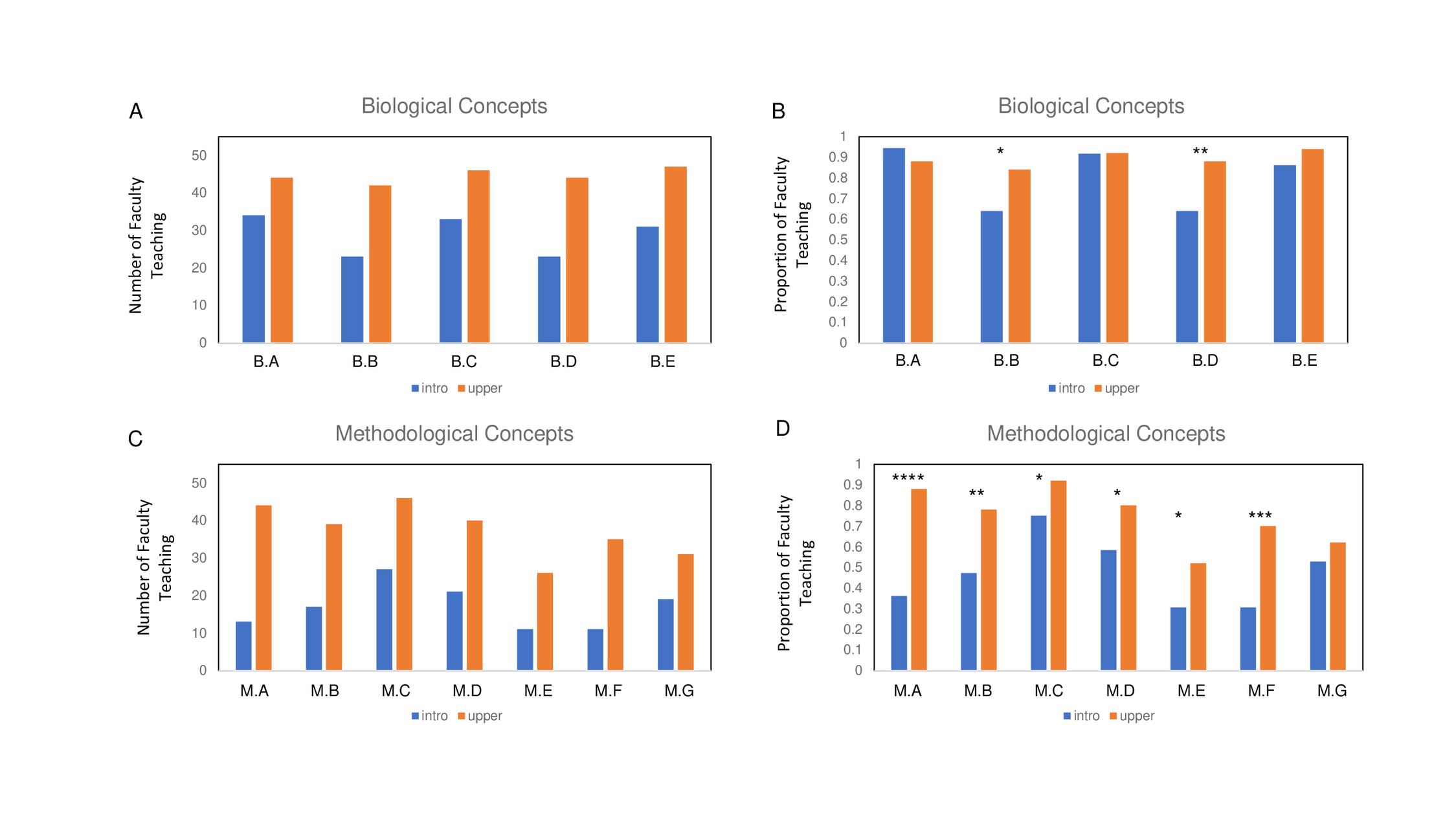

Supplement: S4 Fig — Initial concepts are provided in S1 Table. All biological concepts were taught in at least some genomics courses, but they were taught more frequently at the upper level (A). When normalized to the number of faculty teaching any genomics content at the introductory or upper level, only biological concepts A.B and A.D were taught in a significantly larger proportion of the upper-level courses (B). All methodological concepts were taught in at least some genomics courses, but they were taught more frequently at the upper level (C). Methodological concepts were also taught in a significantly higher proportion of the upper-level courses when normalized to the number of courses taught, except for concept M.G (D).* p<0.05, ** p<0.01, *** p <0.001, **** p<0.0001 by comparison of two proportions Z-test. (TIF) [file pone.0313124.s005.tif]
