# Supplementary material for: A genomics learning framework for undergraduates
Source: PLoS One. 2025 Jan 9;20(1):e0313124. doi: 10.1371/journal.pone.0313124 (PMC11717232; doi:10.1371/journal.pone.0313124)
Supplement: Attachment — Submitted filename: Response to Reviewers.docx [file pone.0313124.s016.docx]

Response to reviewers

PONE-D-24-14148

A genomics learning framework for undergraduates

*1. Please ensure that your manuscript meets PLOS ONE's style requirements, including those for file naming. The PLOS ONE style templates can be found at*

[*https://journals.plos.org/plosone/s/file?id=wjVg/PLOSOne_formatting_sample_main_body.pdf*](https://nam11.safelinks.protection.outlook.com/?url=https%3A%2F%2Fjournals.plos.org%2Fplosone%2Fs%2Ffile%3Fid%3DwjVg%2FPLOSOne_formatting_sample_main_body.pdf&data=05%7C02%7Clreed1%40ua.edu%7C004849b0faa14b67510508dcab390497%7C2a00728ef0d040b4a4e8ce433f3fbca7%7C0%7C0%7C638573508784318801%7CUnknown%7CTWFpbGZsb3d8eyJWIjoiMC4wLjAwMDAiLCJQIjoiV2luMzIiLCJBTiI6Ik1haWwiLCJXVCI6Mn0%3D%7C0%7C%7C%7C&sdata=Z8afIupeshjRHiHBUr6hCQQri2bKte%2FJwmzqIHBB0lI%3D&reserved=0) *and*

[*https://journals.plos.org/plosone/s/file?id=ba62/PLOSOne_formatting_sample_title_authors_affiliations.pdf*](https://nam11.safelinks.protection.outlook.com/?url=https%3A%2F%2Fjournals.plos.org%2Fplosone%2Fs%2Ffile%3Fid%3Dba62%2FPLOSOne_formatting_sample_title_authors_affiliations.pdf&data=05%7C02%7Clreed1%40ua.edu%7C004849b0faa14b67510508dcab390497%7C2a00728ef0d040b4a4e8ce433f3fbca7%7C0%7C0%7C638573508784331971%7CUnknown%7CTWFpbGZsb3d8eyJWIjoiMC4wLjAwMDAiLCJQIjoiV2luMzIiLCJBTiI6Ik1haWwiLCJXVCI6Mn0%3D%7C0%7C%7C%7C&sdata=Yiv2lp0DVYL8tjbyEwJXmj0S9xu0j3kp8%2B9OyZ5X%2FxA%3D&reserved=0)

We examined these two links and modified the manuscript accordingly.

*2. Please provide additional details regarding participant consent. In the ethics statement in the Methods and online submission information, please ensure that you have specified what type you obtained (for instance, written or verbal, and if verbal, how it was documented and witnessed). If your study included minors, state whether you obtained consent from parents or guardians. If the need for consent was waived by the ethics committee, please include this information.*

Participants were informed of the nature of the survey at the beginning of the survey. They could leave the survey at any time without penalty. Thus, completion of the survey was implicit consent. Moreover, the survey was subjected to IRB review at both Georgetown University and Washington University in St. Louis and was deemed exempt at both institutions.

*3. We note that the grant information you provided in the ‘Funding Information’ and ‘Financial Disclosure’ sections do not match.*

*When you resubmit, please ensure that you provide the correct grant numbers for the awards you received for your study in the ‘Funding Information’ section.*

When we began this work, the Genomics Education Alliance was supported by an REU grant from the National Science Foundation (Grant # 1827130 from the Division of Biological Infrastructure [DBI]). However, the grant expired in 2022. Thus, the final stages of this work were not supported by any grant. Please advise as to the best way to report this information.

*4. Thank you for stating the following in the Acknowledgments Section of your manuscript:*

*"The efforts of the Genomics Education Alliance were supported by the National Science Foundation DBI 1827130."*

*We note that you have provided funding information that is not currently declared in your Funding Statement. However, funding information should not appear in the Acknowledgments section or other areas of your manuscript. We will only publish funding information present in the Funding Statement section of the online submission form.*

*Please remove any funding-related text from the manuscript and let us know how you would like to update your Funding Statement. Currently, your Funding Statement reads as follows:*

*"The author(s) received no specific funding for this work."*

Funding information has been removed from the Acknowledgments section.

*Please include your amended statements within your cover letter; we will change the online submission form on your behalf.*

*5. We note that you have included the phrase “data not shown” in your manuscript. Unfortunately, this does not meet our data sharing requirements. PLOS does not permit references to inaccessible data. We require that authors provide all relevant data within the paper, Supporting Information files, or in an acceptable, public repository. Please add a citation to support this phrase or upload the data that corresponds with these findings to a stable repository (such as Figshare or Dryad) and provide and URLs, DOIs, or accession numbers that may be used to access these data. Or, if the data are not a core part of the research being presented in your study, we ask that you remove the phrase that refers to these data.*

The statement has been removed as these data are not a core part of the study. What we meant by this is we performed the matchup as in Table 2 and the Supplementary Tables, but there were few places where these alternate inventories matched with ours, which is essentially how we report this information now. We agree that the statement “data not shown” is superfluous in these instances.

*6. Please include your tables as part of your main manuscript and remove the individual files. Please note that supplementary tables (should remain/ be uploaded) as separate ""supporting information"" files.*

We have added Tables 1 and 2 to the main manuscript.

*7. Please review your reference list to ensure that it is complete and correct. If you have cited papers that have been retracted, please include the rationale for doing so in the manuscript text, or remove these references and replace them with relevant current references. Any changes to the reference list should be mentioned in the rebuttal letter that accompanies your revised manuscript. If you need to cite a retracted article, indicate the article’s retracted status in the References list and also include a citation and full reference for the retraction notice.*

We have reviewed the references. None of the papers have been retracted.

*[Note: HTML markup is below. Please do not edit.]*

*Reviewers' comments:*

*Reviewer's Responses to Questions*

*Comments to the Author*

*1. Is the manuscript technically sound, and do the data support the conclusions?*

*The manuscript must describe a technically sound piece of scientific research with data that supports the conclusions. Experiments must have been conducted rigorously, with appropriate controls, replication, and sample sizes. The conclusions must be drawn appropriately based on the data presented.*

*Reviewer #1: Yes*

*Reviewer #2: Yes*

*________________________________*

*2. Has the statistical analysis been performed appropriately and rigorously?*

*Reviewer #1: Yes*

*Reviewer #2: Yes*

*________________________________*

*3. Have the authors made all data underlying the findings in their manuscript fully available?*

*The PLOS Data policy<*[*http://www.plosone.org/static/policies.action#sharing*](http://www.plosone.org/static/policies.action#sharing)*> requires authors to make all data underlying the findings described in their manuscript fully available without restriction, with rare exception (please refer to the Data Availability Statement in the manuscript PDF file). The data should be provided as part of the manuscript or its supporting information, or deposited to a public repository. For example, in addition to summary statistics, the data points behind means, medians and variance measures should be available. If there are restrictions on publicly sharing data—e.g. participant privacy or use of data from a third party—those must be specified.*

*Reviewer #1: Yes*

*Reviewer #2: Yes*

*________________________________*

*4. Is the manuscript presented in an intelligible fashion and written in standard English?*

*PLOS ONE does not copyedit accepted manuscripts, so the language in submitted articles must be clear, correct, and unambiguous. Any typographical or grammatical errors should be corrected at revision, so please note any specific errors here.*

*Reviewer #1: Yes*

*Reviewer #2: Yes*

*________________________________*

*5. Review Comments to the Author*

*Please use the space provided to explain your answers to the questions above. You may also include additional comments for the author, including concerns about dual publication, research ethics, or publication ethics. (Please upload your review as an attachment if it exceeds 20,000 characters)*

*Reviewer #1: The manuscript is well conceived and clearly written. The authors have created a thorough list of concepts for genomics that will be very helpful to educators. I have only a few minor suggestions.*

*The list of methodological concepts mentions genome sequencing and metagenomics, but does not mention RNA-Seq. Adding a concept (perhaps MC.II.C) about RNA-Seq as a way to gain information about which portions of the genome are expressed and to compare expression under different conditions/cell types would make the methodological concepts more complete. However, since that would require redoing all of the comparisons to other inventories, adding an explicit mention of RNA-Seq to BC.IV.C would probably suffice.*

Thank you for noticing this issue.  We have added MC.II.C “Sequencing of populations of RNA molecules (e.g., RNA-Seq) is a way to gain information about variation in which portions of the genome are expressed across individuals, environmental conditions, tissues, and cell types.” This addition has minimal impact on the concept inventory comparisons and the appropriate updates have been made.

*In concept EC.II.G Should it be “patent” law instead of “patient” law?*

Yes!  We have corrected this typo.  Thank you for catching it.

*Table S4: The Course Source Genetics Core Concepts Headings need to be wrapped so they are completely visible*

These headings have been adjusted for visibility.

*Reviewer #2: The manuscripts presents the development of a genomics concept list that provides an updated set of topics and subjects that should be considered in development and instruction of genomics courses. The development and design of this concepts list was thorough and comprehensive, with valuable input by appropriate faculty. The authors provide detailed comparison with existing lists and inventories to demonstrate that there is a niche and need for this updated concept list. Further, authors mention how the new concept list will be useful in development of genomics curriculum and assessment.*

*The manuscript is organized well, methodology is clearly stated and the information is clearly presented.*

*Specific areas to review:*

*The supplementary figures were not made available. The supplementary figure legends were provided and the main result of the figures was presented in the text. However, the figures were not available for review.*

We apologize for this oversight. The supplementary figures were part of the same file containing Figure 1 but was not labeled adequately. All figures are now available as individual tiff files.

*Line 79: PHAGES is acronym, should be capitalized and hyphenated*

This correction has been made.

*Line 147: A short description of the genomic educators would be helpful, for instance what level courses (generally lower/upper level) and/or what type of institutions (2 yr, 4 yr, grad?) do they represent? Are they the n=15 BIOME members from line 203?*

We added the phrase at the end line 148 *“.....a combination of 2-year, 4-year, and research intensive institutions, both public and private”.*  The founding members of GEA and others who attended our workshops and virtual meetings as we developed these genomics concepts included four from community colleges, five from private, 4-year residential colleges, and the remainder from public or private research-intensive (R1 or R2) institutions. We considered both introductory and advanced undergraduate curricula. The 15 BIOME members provided additional feedback during the 2020 virtual workshop before we surveyed a larger group of educators as described.

*Line 148-151: how many faculty were surveyed*

61, this has been added to the text

*Table S1: “rekecting” in B.A. section*

This has been edited to say “reflecting”.  Thank you for noticing the typo.

*Paragraph 282-296: Consider: It could be helpful to point out some of the examples where the genomics concepts do not overlap the existing inventories, indicating support for the newly developed genomics concepts list. At least for the comparison to bioinformatics inventories (line 293), what was missing from existing bioinformatics inventories that this new genomics concept list offers? To a reader this would provide a quick indication of how, in addition to V&C and other inventories, the presented genomics concepts provides an updated and more complete set of concepts relating to instructing genomics.*

We have chosen not to include examples in the text given that the places where overlap is not seen are given in the Tables (both Table 2 and the Supplementary Tables).

*________________________________*

*6. PLOS authors have the option to publish the peer review history of their article (what does this mean?<*[*https://journals.plos.org/plosone/s/editorial-and-peer-review-process#loc-peer-review-history*](https://journals.plos.org/plosone/s/editorial-and-peer-review-process#loc-peer-review-history)*>). If published, this will include your full peer review and any attached files.*

*If you choose “no”, your identity will remain anonymous but your review may still be made public.*

*Do you want your identity to be public for this peer review? For information about this choice, including consent withdrawal, please see our Privacy Policy<*[*https://www.plos.org/privacy-policy*](https://www.plos.org/privacy-policy)*>.*

*Reviewer #1: No*

*Reviewer #2: No*

*________________________________*
